# Numerical Analysis of Piezoelectric Active Repair in the Presence of Frictional Contact Conditions

**DOI:** 10.3390/s130404390

**Published:** 2013-04-02

**Authors:** Andrea Alaimo, Alberto Milazzo, Calogero Orlando, Antonio Messineo

**Affiliations:** 1 Faculty of Engineering and Architecture, University of Enna Kore, Cittadella Universitaria 94100, Enna, Italy; E-Mails: calogero.orlando@unikore.it (C.O.); antonio.messineo@unikore.it (A.M.); 2 Dipartimento di Ingegneria Civile Ambientale Aerospaziale dei Materiali, University of Palermo, Viale delle Scienze 90128, Palermo, Italy; E-Mail: alberto.milazzo@unipa.it

**Keywords:** active repair, piezoelectric actuator, boundary element method, spring model, frictional contact

## Abstract

The increasing development of smart materials, such as piezoelectric and shape memory alloys, has opened new opportunities for improving repair techniques. Particularly, active repairs, based on the converse piezoelectric effect, can increase the life of a structure by reducing the crack opening. A deep characterization of the electromechanical behavior of delaminated composite structures, actively repaired by piezoelectric patches, can be achieved by considering the adhesive layer between the host structure and the repair and by taking into account the frictional contact between the crack surfaces. In this paper, Boundary Element (BE) analyses performed on delaminated composite structures repaired by active piezoelectric patches are presented. A two-dimensional boundary integral formulation for piezoelectric solids based on the multi-domain technique to model the composite host damaged structures and the bonded piezoelectric patches is employed. An interface spring model is also implemented to take into account the finite stiffness of the bonding layers and to model the frictional contact between the delamination surfaces, by means of an iterative procedure. The effect of the adhesive between the plies of piezoelectric bimorph devices on the electromechanical response is first pointed out for both sensing and actuating behavior. Then, the effect of the frictional contact condition on the fracture mechanics behavior of actively repaired delaminated composite structures is investigated.

## 1. Introduction

The increasing development of smart materials, such as piezoelectric and smart memory alloys, has opened new opportunities for practical and useful engineering applications. Focusing on piezoelectric materials, they allow the design of effective sensors and actuator devices, by means of the direct and converse piezoelectric effect [1], which, nowadays, are widely used in the framework of Structural Health Monitoring (SHM) [2–5], Active Vibration Control [6,7] as well as Energy Harvesting [8,9].

Recently, the use of piezoelectric actuators has been also proposed and studied, by both numerical and experimental analyses, for applications in so-called active repair technology [10–16]. This repairing technique, if obtained by means of piezoelectric devices, is based on the converse piezoelectric effect, according to which the strain induced by an applied electric field across the piezoelectric patch can help the structure to oppose the external load and, consequently, to reduce the criticality of a damage [15,17]. It thus represents improved repair methodology alternatives to the conventional methods, like bonded or riveted passive patches, whose limitations have been widely reviewed [18]. Actually, active repair technology and Structural Health Monitoring could effectively be implemented in the framework of the so-called closed-loop smart structures, where components are able to sense, diagnose and actuate in order to perform their functions [19].

In spite of its attractiveness, active repair is a very difficult problem involving both design and technological aspects. From the design point of view, active repairs could be, in fact, arranged by bonding piezoelectric actuators to the host damaged structures and, in order to gain full advantage of the active repair technology, a deep characterization of both the electromechanical and fracture mechanics behavior of the actively repaired damaged structures is needed. In fact, the complex active repair mechanism, which stems from the strain induced by the piezoelectric actuator to the damaged structure by means of the bonding layer, can be fully understood through efficient numerical tools, taking into account both the adhesive between the bonded layers and the frictional contact condition between the crack or delamination surfaces. With this aim, Liu [17,20] has recently proposed a two-dimensional finite element analysis to study the active repair for cracked structures by using multi-layered piezoelectric patches, while Duan *et al.* [21] have applied the finite element method to study actively repaired delaminated beams. The effect of the adhesive between the host damaged structure and the piezoelectric patch has been studied by Alaimo *et al.* [15,16], where the boundary element method has been used to characterize the static and dynamic electromechanical behavior of isotropically damaged structures actively repaired with bonded piezoelectric patches.

As already mentioned, a more accurate characterization of the repairing mechanism of piezoelectric active patches can be achieved through the modeling of the frictional contact between the crack surfaces. The interaction between two crack surfaces in contact, in fact, gives rise to a sliding resistance, depending on the normal and tangential tractions at the interface. It is evident that the modeling of such a situation can lead to a more accurate prediction of the electromechanical response of the actively repaired damaged structure [17]. Moreover, when dealing with a multilayered piezoelectric actuator, the bonding layer between the piezoelectric plies can deeply affect the actuating performance of the repair.

Thus, the present paper deals with the analysis of the electromechanical response of piezoelectric active patches applied on delaminated composite structures, accomplished through a boundary element code [22–24], which allows one to take into account the adhesive between the piezoelectric plies, used to arrange the multilayered patch, and the Coulomb's frictional contact between the crack surfaces. This is accomplished by means of multi-domain techniques implemented with an interface spring model, which allows one to model the Coulomb's frictional contact through a suitable iterative procedure. On this basis, the proposed model and the associated results have to be considered as a preliminary characterization useful to effectively set forward future experimentation and to get insight into the active repair mechanism that will allow one to develop proper autonomous repairing control systems. Moreover, the numerical analyses can provide useful data for the practical implementation of the technological aspects that, however, are out of the scope of the present paper.

The content of the paper is arranged as follows: the Multi-domain Boundary Element formulation and the spring interface iterative procedure for the modeling of the Coulomb's frictional contact are discussed in Section 2; The Modified Crack Closure Integral (MCCI) [25], used to compute the total Energy Release Rate (ERR) *G_T_* and the phase angle Ψ, is introduced in Section 3; lastly, numerical results are reported and discussed in Section 4. More particularly, validation analysis on the Coulomb's frictional contact is first discussed in order to show the effectiveness of the implemented frictional contact conditions based on the modified interface spring model; then, the effect of the adhesive between piezoelectric plies of the multilayered patch is investigated for both sensing and actuating devices with the aim of gaining insight on the importance of modeling the bonding layer for an effective characterization of the electromechanical response of the piezoelectric patch; lastly, original numerical results, characterizing the fracture mechanics behavior of a composite drop-ply delaminated structure in terms of the energy release rate reduction and phase angle variation, are presented to highlight the effect of frictional contact on the repair performances.

## 2. Multi-Domain Boundary Element Method (BEM) and Coulomb's Friction Modeling Strategy

The Boundary Integral formulation is developed for a two-dimensional piezoelectric domain, Ω, with boundary, ∂Ω, lying in the *x_1_x_2_* plane under the hypothesis of generalized plain strain elasticity and in-plane electrostatic. For the sake of conciseness, the governing equations of the problem are not reported in the present paper, but can be found in reference [15]. Considering a particular electroelastic state defined by the generalized displacement field, **U***_j_*, associated with the concentrated generalized body forces acting in an infinite domain and applied at the point, *P*_0_, F_j_ = ***c****_j_δ*(*P, P_0_*), where *δ* is the Dirac's function and **c***_j_* is the load intensity, the reciprocity theorem for this particular and the actual electroelastic states leads to an analogon of the Somigliana identity for the electromechanical problem [24]:

(1) $${{\text{c}}_{j}}^{T}\text{U}\;(P_{0})+\int_{\partial \Omega }\left({{\text{T}}_{j}}^{T}\text{U}-{{\text{U}}_{j}}^{T}\text{T}\right)\;d\partial \;\Omega =\int_{\Omega }{{\text{U}}_{j}}^{\text{T}}\text{F}d\;\Omega$$

where **T***_j_* are the generalized tractions associated to **U***_j_*. By using four independent fundamental solutions [24], the mechanical displacements and the electric potential at point, *P_0_*, can be expressed in terms of the generalized displacements and tractions on the boundary of the body In matrix form, the boundary integral equations, obtained by collocating the point, *P_0_*, to the boundary ∂Ω [22], are given by:

(2) $$\text{c}*\text{U}\;(P_{0})+\int_{\partial \Omega }(\text{T}*\text{U}-\text{U}*\text{T})\;d\partial \;\Omega =\int_{\Omega }\text{U}*\text{F}d\;\Omega$$

where the kernels, U* and T*, and the matrix, c*, are defined as by Alaimo *etal.* [15]. The numerical model of the boundary integral formulation is obtained by means of the Boundary Element Method (BEM), which for a homogeneous body, involves the division of the body boundary into boundary elements over which the generalized displacements, **U**, and the associated boundary tractions, **T**, are interpolated in terms of their nodal values, *δ* and **P**, by means of proper shape functions. With this approximation, the discrete expression of the solving equations for piezoelectric media are thus obtained and written as:

(3) Hδ+GP=0

Equation (3) represents a linear algebraic resolving system expressed in terms of generalized displacement and traction nodal values, *δ* and **P**, respectively, which coupled with the electromechanical boundary conditions, provide the solution of the problem for a single domain.

The modeling of the laminated structure, as well as the assembling between the host structure and the piezoelectric active patch is then obtained by means of the multi-domain approach [22,24]. It is implemented by writing the system of equations, Equation (3), for each of the *N* homogeneous sub-region as:

(4) $$\begin{array}{cc} {\text{H}}^{\left(k\right)}\;\delta^{\left(k\right)}+{\text{G}}^{\left(k\right)}\;{\text{P}}^{\left(k\right)} & k=1,2\ldots ,N \end{array}$$

**Figure 1.**
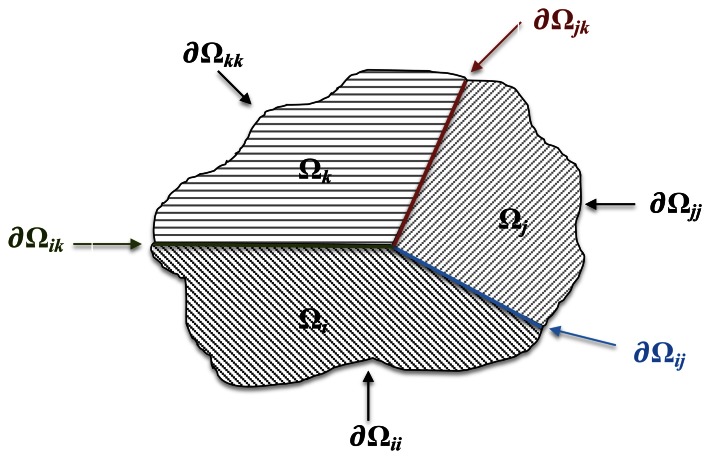
Multi-domain assembling.

**Figure 2.**
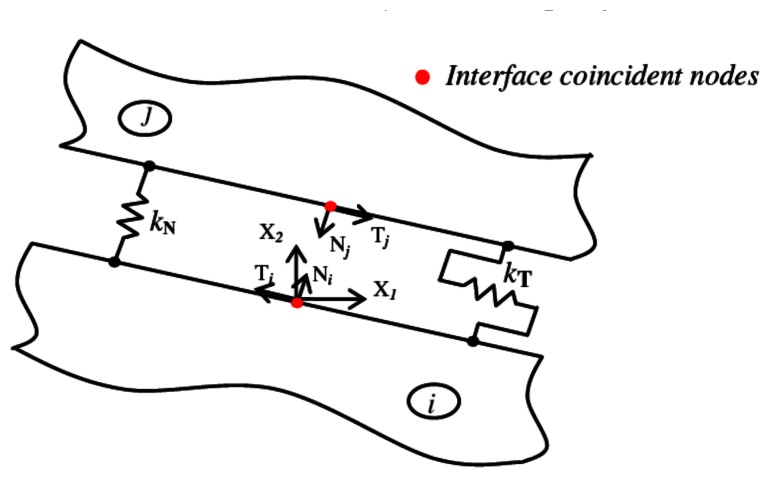
Interface local reference system and Spring Model.

The global system of equations pertaining to the overall assembled structure is then obtained by applying the compatibility and equilibrium conditions along all the sub-region interfaces:

(5) $$\begin{array}{cc} \delta_{\partial \Omega_{ij}}^{\left(i\right)}-\delta_{\partial \Omega_{ij}}^{\left(j\right)}=\Delta \;\delta^{ij} & i=1,\ldots ,N-1\\ {\text{P}}_{\partial \Omega_{ij}}^{\left(i\right)}=-{\text{P}}_{\partial \Omega_{ij}}^{\left(j\right)} & j=i+1,\ldots ,N \end{array}$$

where the subscript, *∂*Ω*_ij_*, indicates quantities associated with the nodes belonging to the interface between the *i*th and *j*th sub-regions; see Figure 1. Equation (5) allows one to assemble heterogeneous sub-regions by considering both rigidly and elastic connections among them. More particularly, the first condition is obtained by enforcing to zero the displacement jump, Δ*δ*^ij^. Conversely, the elastic interface conditions are addressed by means of an interface Spring Model [15], which allows one to model the adhesive ply as a zero thickness elastic layer characterized by normal and tangential compliance constants, *k_N_* and *k_T_*, with respect to a local reference system centered at each node of the *i*th domain interface boundary, shown in Figure 2.

More particularly, by considering the local reference system of Figure 2, the spring model is implemented by expressing the normal and tangential components of the interface mechanical displacement jumps, $\Delta \;\delta_{N}^{\left(ij\right)}$ and $\Delta \;\delta_{T}^{\left(ij\right)}$, as a function of the normal and tangential components of the nodal mechanical tractions:

(6) $$\begin{array}{ccc} \Delta \;\delta_{N}^{\left(ij\right)}=k_{N}{\text{P}}_{N}^{\left(i\right)} & \text{with} & \Delta \;\delta_{N}^{ij}=\Delta \;\delta_{N}^{\left(j\right)}-\;\delta_{N}^{\left(i\right)}\\ \Delta \;\delta_{T}^{\left(ij\right)}=k_{T}{\text{P}}_{T}^{\left(i\right)} & \text{with} & \Delta \;\delta_{T}^{ij}=\Delta \;\delta_{T}^{\left(j\right)}-\;\delta_{T}^{\left(i\right)} \end{array}$$

The spring interface, expressed by Equation (6), allows for the modeling of the bonding layer between the host structure and the piezoelectric patch by linking the aforementioned interface compliance coefficients, *k_N_* and *k_T_*, to the material properties of the adhesive through a strategy discussed by Alaimo *et al.* [15]. The modeling of crack contact problems, governed by the classical Coulomb's law of friction, is, instead, achieved by means of a trial and error procedure that iteratively sets the compliance coefficients, *k_N_* and *k_T_*, depending on the modes of contact [26]. More in detail, the iterative procedure firstly accomplishes the detection of the potential contact area, avoiding, at the same time, the unphysical material interpenetration corresponding to negative values of $\Delta \delta_{N}^{ij}$.

**Figure 3.**
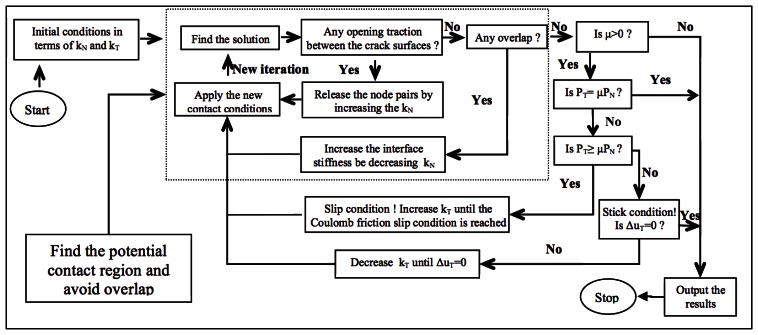
Flow chart of the frictional contact iterative procedure.

The aim at issue is achieved by varying the *k_N_* coefficients according to the sign of the normal traction components. Once the potential contact area is determined, the iterative procedure starts checking the friction contact modes. According to Coulomb's law of friction, if **P***_T_* is less than *μ***P***_N_*,*μ* being the friction coefficient, then the tangential compliance coefficient, *k_T_*, is decreased until the stick condition is assured. On the other hand, if **P***_T_* exceeds *μ***P***_N_*, the node pairs are allowed to sleep, and then, the coefficient, *k_T_*, is incrementally increased until the Coulomb friction slip condition is reached. A schematic of the iterative procedure implemented is shown in Figure 3.

## 3. Modified Crack Closure Integral Technique

In this paper, the total ERR, *G_T_*, and the mode-mix phase angle, Ψ [27,28], are used as fracture parameters to fully characterize cracks at a bi-material interface. The former fracture parameter is obtained as the sum of the ERR components associated with the modes of fracture, which with reference to the local frame centered at the crack tip, can be expressed as:

(7) $$\begin{array}{c} G_{I}=\underset{\Delta \to 0}{\lim}\frac{1}{2\Delta }\;\int_{0}^{\Delta }t_{N}\;(\Delta -r)\Delta u_{N}\;(r)dr\\ G_{II}=\underset{\Delta \to 0}{\lim}\frac{1}{2\Delta }\;\int_{0}^{\Delta }t_{T}\;(\Delta -r)\Delta u_{T}\;(r)dr \end{array}$$

where Δ*u_i_, i* = {*N*, T}, are the displacement jump components at a distance, *r*, behind the crack front, along the opening and sliding directions respectively, *t_i_* are the normal and shearing traction components and Δ is an infinitesimal crack extension. The latter fracture parameter, Ψ, is the sliding to opening mode-mix phase angle, whose computation is also mandatory for bi-material interface fracture mechanics, since the total ERR *G_T_* by itself does not allow for the identification of the critical condition [29].

Thus, the mode-mixed phase angle, Ψ, is computed, under the assumption of negligible oscillatory behavior of the crack front stress and displacement fields [30] by the ERR components, as:

(8) $$\psi =\tan^{-1}\;\left(\sqrt{\frac{G_{II}}{G_{I}}}\right)$$

The modified crack closure integral MCCI technique is thus used for the computation of the total ERR, *G_T_*, and of the mode-mixed phase angles, Ψ. It represents a robust method, since a reasonable accuracy in the computation of the fracture mechanics parameters can be obtained, even without additional mesh refinement or high-order/singular crack tip elements, by both Finite and Boundary Element Procedures [25,31,32]. Moreover, by using the MCCI technique, no further computations of stresses and displacements are required. In particular, for a given laminate showing an initial delamination length, *a*, the MCCI assumes that, as the delamination propagates by a length, Δ [33]:

(i) the energy released is identical to the work required to close the crack;
(ii) Δ being small enough, the crack extends in a self-similar manner.

The second assumption, in particular, implies that the stress field does not change as the crack extends from *a* to *a* + Δ, and thus, a single analysis suffices all data needed for Irwin's integral computation. As a consequence, the boundary element mesh is built in such a way as to be symmetric across the crack front in order to numerically favor the self-similar extension assumption. Moreover, the BE mesh size is small enough to catch the limiting nature of Irwin's integral Equation (7) in a finite counterpart as well as possible. In order to perform the integrals defined in Equation (7), regular elements should be used, since the product of stresses and displacements represents energy that is finite everywhere in the body, including the region next to the crack front [34]. Thus, linear shape functions are used in the present work, and the smoothing scheme proposed by Maiti *et al.* [35] is implemented.

**Figure 4.**
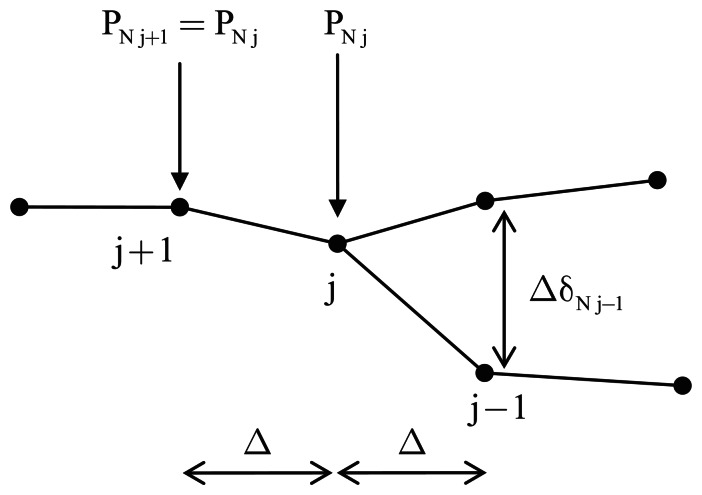
Smoothing scheme linear element.

The traction variation along the boundary element, including the crack tip, identified by node *j* in Figure 4, is assumed consistent with the displacement variation. Thus, accordingly to Maiti *etal.* [35], the constant value of the traction over the crack tip element is assumed to be equal to that at node *j*. Thus, with reference to Figure 4, the integral definition of the ERR components, Equation (7) specifies it as:

(9) $$\begin{array}{cc} G_{I}=\frac{\delta_{N_{j-1}}P_{N_{j}}}{2} & G_{II}=\frac{\delta_{T_{j-1}}P_{T_{j}}}{2} \end{array}$$

## 4. Numerical Results and Discussion

In the present section, the numerical results obtained are discussed. More particularly, Coulomb's friction modeling strategy as proposed is first validated through the analysis of a flat punch over an elastic foundation previously studied by Man [26]. Then, the effect of the adhesive between the piezoelectric plies of a bimorph device on the sensing and actuating electromechanical performances is pointed out. Lastly, the active repair mechanism of a drop-ply delaminated composite structure repaired through a multi-layered PZT-4 active patch is investigated by taking the frictional contact conditions into account.

### 4.1. Coulomb's Friction Modeling Strategy Validation

**Figure 5.**
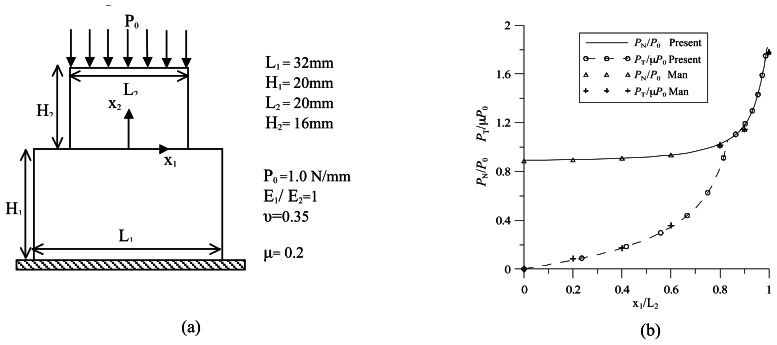
(**a**) Punch on an elastic foundation configuration; (**b**) normalized contact traction components.

Coulomb's friction modeling strategy as proposed in the present paper is validated through the analysis of a flat punch over an elastic foundation previously studied by Man [26]. The geometry, the boundary conditions, as well as the material properties of the two bodies and the friction coefficient are summarized in Figure 5(a). The results obtained are plotted in Figure 5(b) in terms of the normal and tangential tractions at the interface between the two bodies. Both traction components are normalized and, due to symmetry, are plotted along half the contact region. More particularly, the normal traction component has been normalized with respect to the applied load, *P*_o_, while the tangential one, with respect to *μP_0_*. As already highlighted by Man [26], at *x*_1_*/L*_2_ = 0.8, the transition point from stick to slip condition can be found, and good agreement with the results found in the literature is evidenced.

### 4.2. Interface Adhesive Modeling in Piezoelectric Bimorph Sensor/Actuator Device

In order to point out the effect of the adhesive between the piezoelectric plies of bimorph devices to be used as sensors and actuators, two different analyses of a piezoelectric series bimorph are performed for both sensing and actuating functions. The geometry of the piezoelectric bimorph devices has been chosen, since 3-D electromechanical data for the perfect bonding condition were available in the literature [36], thus allowing for the validation of the piezoelectric BEM formulation and for pointing out the effect of the finite bonding stiffness on the electromechanical response with respect to the validated perfect bonding results.

**Figure 6.**
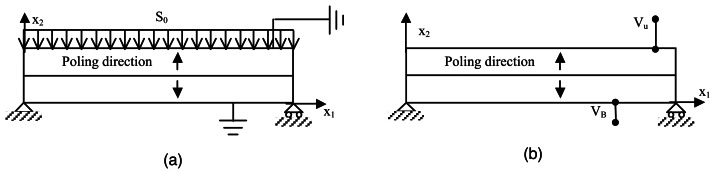
Series bimorph configuration as sensor (**a**) and actuator (**b**).

The first configuration deals with the piezoelectric device used as a sensor in a closed circuit; see Figure 6(a). The length of the bimorph is *L* = 25 *mm*, and a slender ratio, *L*/*h* = 10, is considered. Moreover, the boundary conditions and the material properties are those taken from Fernandes et al. [36]

**Figure 7.**
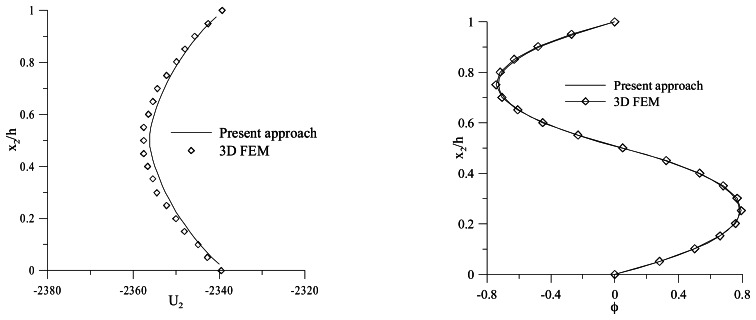
Through-the-thickness vertical displacement *U_2_* and electric potential *ϕ* distribution.

Figure 7 shows the through-the-thickness normalized vertical displacement and electric potential distributions at *x*_1_ = *L*/2 in the case of a perfect bonded interface. The results are compared to those obtained by the finite element analysis performed for the full 3-D model by Fernandes *et al.* [36].

**Figure 8.**
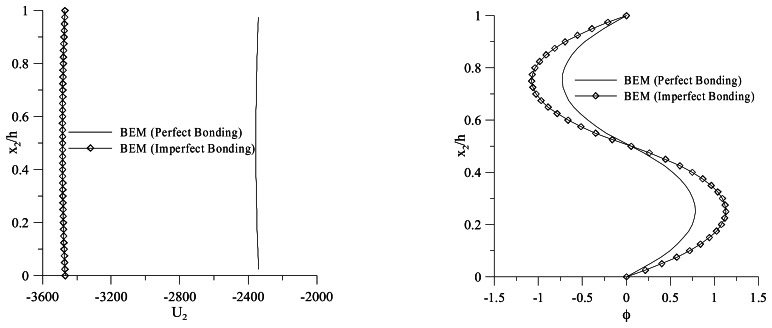
Vertical Displacement Boundary Element Method (BEM), Electric Potential BEM (perfect/imperfect bonding).

Figure 8 shows the vertical displacement distribution obtained for both perfect and imperfect bonding conditions. In particular, the imperfect bonding conditions are set to model a 0, 1 mm epoxy adhesive layer. A 32% increment of the deflection in the case of imperfect bonding, due to the softening of the bimorph, can be observed. This leads to a change of the through-the-thickness electric potential distribution, as shown in Figure 8. The modeling of the adhesive layer is then of significant importance in order to correctly interpret the output of a bimorph used with a sensing function.

**Figure 9.**
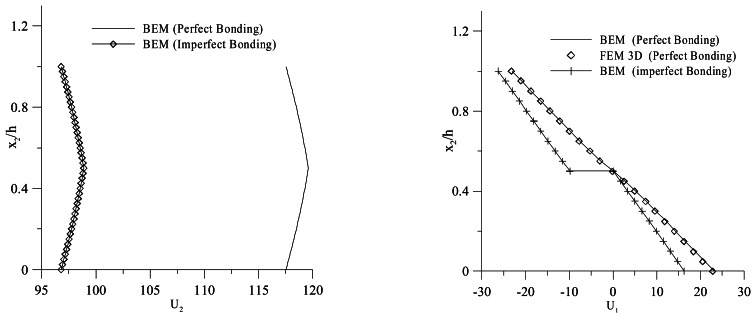
Through-the-thickness vertical displacement and longitudinal displacement.

To point out the effect of the adhesive layer on the actuating performances of a piezoelectric bimorph with a series arrangement, in the second configuration analyzed, an electric potential is applied on the top and bottom faces of the device, *V_B_* = −50 V at *x*_2_ = 0 and *V_U_* = 50 V at *x*_2_ = *h*, as shown in Figure 6(b). Figure 9 shows the through-the-thickness normalized vertical and longitudinal displacement. The softening of the bimorph actuator, due to the adhesive layer, can be pointed out by observing a 17% reduction of the through-the-thickness deflection. Thus, the results show the need of modeling the bonding layer effects to accurately predict the actuating input voltage.

### 4.3. Actively Repaired Drop-Ply Delaminated Structure with Frictional Contact Conditions

**Figure 10.**
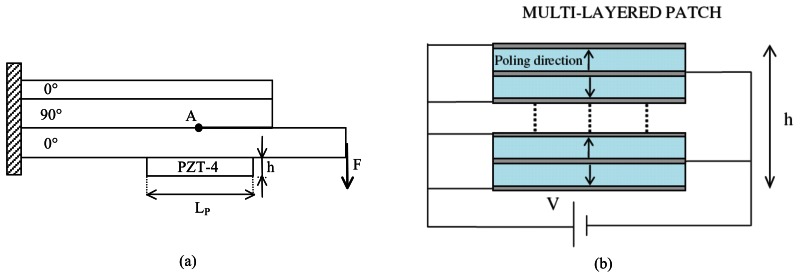
(**a**) Repaired structure configuration; (**b**) PZT-4 multi-layered circuital scheme.

The analysis discussed in the present section deals with a drop-ply delaminated composite structure repaired through a multi-layered PZT-4 active patch, as shown in Figure 10(a). The geometry and the boundary conditions considered for the host damaged composite structures, as well as the material properties of the graphite/epoxy system are those used by Narayan et al. [25].

A multi-layered piezoelectric patch having length, *L_P_* = 20.4 *mm*, and height, *h* = 1.5 *mm*, whose circuital scheme is shown in Figure 10(b), is bonded on the lower ply in a central position with respect to the crack tip. The active repair is then obtained by stacking 10 piezoelectric layers, whose material properties are those considered by Alaimo *et al.* [15]. This patch configuration has been selected, since it provides a strain per applied voltage larger than other configurations [15]. Moreover, according to the bonding modeling strategies employed in the present paper, the equivalent compliance interface coefficients, corresponding to an epoxy adhesive having thickness, *t_a_* = 0.1 *mm*, are *k_N_* = 1.56 × 10-^5^*m/GPa* and k_T_ = 9.33 × 10-^5^*m/GPa*. The fracture mechanics behavior of the repaired structure is characterized for different values of the friction coefficient, *μ*, in terms of the total Energy Release Rate *G_T_* and of the phase angle, *ψ*.

**Figure 11.**
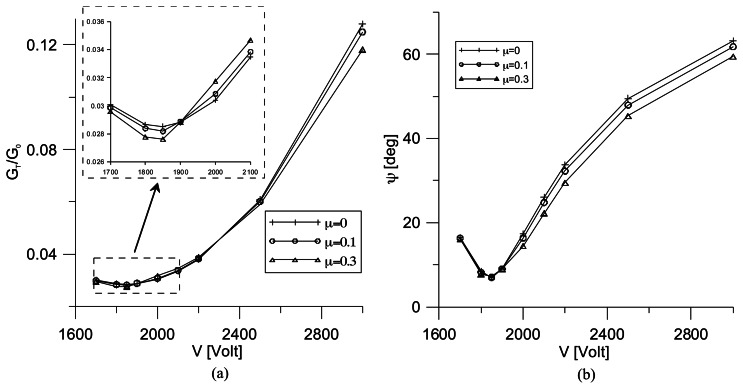
Effect of the friction coefficient on the fracture parameters.

Figure 11 shows the total energy release rate distribution *versus* the applied voltage, *V*, obtained for different values of the friction coefficient, *μ*. The energy release rate is plotted in dimensionless units by dividing Gt to G0, G0 being the total energy release rate characterizing the fracture mechanics behavior of the un-repaired structure. It is worth noting that the computed G0 = 1.43 × 10-4 MPam agrees very well with that found by Narayan et al. [25] through a finite element analysis. From Figure 11, it can be observed that the repair condition for the three different contact configurations analyzed, corresponding to the minimum value of the total energy release rate, is reached at the same value of the repairing voltage, *V_r_* = 1.850 *V*. Thus, it can be firstly concluded that the different frictional contact conditions do not have valuable influence on the repairing voltage value. On the other hand, for a given applied voltage, *V* < *V_r_*, the effect of the frictional contact between the crack surfaces is to improve the repair performance of the active patch till the minimum value of *G_T_* is obtained. The analysis of the post-repair condition, corresponding to *V* > *V_r_*, evidences the same behavior as for *V* < *V_r_*, with the only exception inside the interval 1, 900 < *V* < 2, 200, where the repair performances of the friction contact configuration are worse than the frictionless one. The graph of Figure 11 shows that, once the repair condition is reached, the phase angle, *ψ*, gets its minimum value. Moreover, the effect of friction on the mode mix is to reduce *ψ* and, consequently, the amount of the mode II of fracture with respect to mode I.

## 5. Conclusions

In this paper, BE analyses performed on delaminated composite structures repaired by active piezoelectric patches have been presented. A two-dimensional boundary integral formulation for piezoelectric solids based on the multi-domain technique to model the composite host damaged structures and the bonded piezoelectric patches has been employed. An interface spring model has been also implemented to take into account the finite stiffness of the bonding layers and to model the frictional contact between the delamination surfaces, by means of an iterative procedure. The analyses have shown that the finite stiffness of the bonding layer deeply affects the electromechanical response of piezoelectric devices. On the other hand, it has been pointed out that the frictional contact condition has a negligible effect on the repairing mechanism for the analyzed delaminated structure.
